# Exosomal miR-122-5p for regulation of secretory functions of fibroblasts and promotion of breast cancer metastasis by targeting MKP-2: an experimental study

**DOI:** 10.1080/15384047.2025.2500104

**Published:** 2025-05-04

**Authors:** Yun Lv, Yue Li, Jie Zhou, Xin Liu, Dandan Wang, Dongmei Wang, Dandan Tong, Shuhuai Wang, Hanxiang An, Xinmei Kang

**Affiliations:** a Department of Medical Oncology, Xiang’an Hospital of Xiamen University, School of Medicine, Xiamen University, Xiamen, China; b Department of Medical Oncology, Heze Municipal Hospital, Heze, China; c Department of Ultrasonography, Xiang’an Hospital of Xiamen University, Xiamen, China; d School of medicine, Huaqiao University, Quanzhou, China; e Department of Pathology, Cancer Hospital of Harbin Medical University, Harbin, China; f Xiamen Key Laboratory of Endocrine-Related Cancer Precision Medicine, School of Medicine, Xiamen University, Xiamen, China

**Keywords:** miR-122-5p, exosome, breast cancer, fibroblast, metastasis

## Abstract

Tumor metastasis is a major obstacle for the effective treatment of breast cancer. Some studies showed that exosomes could promote tumor distant metastasis by establishing pre-metastasis niches (PMN). MicroRNAs (miRNAs) in exosomes play a critical role in tumor development and invasion. We aimed to investigate the effects of exosomal miRNAs derived from breast cancer cells on metastasis. MiRNA sequencing and RT-PCR approach were used to screen potential exosomal miRNAs. We compared the levels of serum exosomal miRNAs from breast cancer patients and those from MCF10A/MCF7/MDA-MB-231 cells. We found that differential exosomal miRNAs screened from patients with metastasis have higher expression levels in exosomes secreted by MDA-MB-231 cells. Using miRNA mimics or inhibitors, exosomal miR-122-5p was found to enhance the secretion levels of chemokine MCP-1 and SDF-1 from WI-38 lung fibroblast cells. In vitro luciferase assay and western blot confirmed the targeting of 3’-untranslated region of MKP-2 and suppression of MKP-2 expression by miR-122-5p in WI-38 cells. Treatment of xenograft mice with exosomal miR-122-5p increased the levels of MCP-1 and SDF-1 in serum, and promoted lung metastasis of breast cancer. In conclusion, we identified exosomal miR-122-5p from breast cancer cells that could promote the chemokine secretion of lung fibroblasts, which might facilitate the chemotaxis and colonization of breast cancer cells in lung tissue.

## 1. Introduction

Nearly 12% of patients with a diagnosis of breast cancer eventually develop metastatic disease. The 5-year survival rate is only 26% in metastatic breast cancer patients.^1^ Researchers found that rapidly rising distant-stage rates contributed to high mortality rates in breast cancer women younger than 40 years.^2^ Tumor metastasis is a series of multi-step process. Initially, normal cells mutate into tumor cells, which proliferate and grow rapidly in the primary site. At a certain stage, some tumor cells change the surrounding matrix environment, and penetrate into the humoral circulatory system, where most of the circulating tumor cells (CTCs) are killed or destroyed by immune cells. Less than 0.02% CTCs survive and are transported to the microvascular bed of distant tissues, and finally colonize ectopic tissues.^3^ The specific molecular mechanism of the whole metastasis process has not been well explained up to now. In the process of tumor metastasis, in addition to local signals in the primary tumor microenvironment, tumor cells also form the pre-metastasis niches (PMN) by sending signals to distant pre-metastatic sites, paving the way for the transplantation and growth of circulating tumor cells.^4,5^

Fibroblast is an important component of normal tissue matrix and is mainly involved in the synthesis of extracellular matrix of connective tissue. They can participate in tumor progression by remodeling of the extracellular matrix (ECM). Studies have shown that activated cancer-associated fibroblasts (CAFs) can induce interstitial remodeling, which is of vital significance for the formation of PMN.^6,7^ Fibroblasts can be activated into CAFs by tumor-derived substances. CAFs are highly heterogeneous and secrete multiple cytokines. These secretory proteins were classified as secretory vesicles and secretory particles in the Trans-Golgi network (TGN), which are different vesicle carriers of constitutive secretion and regulatory secretion, respectively.^8^ When the cells are activated, the Golgi apparatus expands and the cell secretion ability is enhanced. Cytokines secreted by CAFs contribute to tumor growth and metastasis. CAFs play pivotal roles in monocyte recruitment and macrophage polarization through monocyte chemotactic protein-1 (MCP-1) and stromal cell-derived factor-1 (SDF-1) in breast cancer.^9^ Exosome-mediated miRNA delivery has been shown to contribute to tumor cell proliferation, metastasis, and therapeutic resistance. Some exosomal miRNAs can promote the expression of mesenchymal-related molecules, change the PMN, and then promote tumor’s progression.^10,11^ One study showed that exosomes secreted by human high-metastatic breast cancer cells carry miR-200, which can be taken up by low-metastatic cells, and then through promoting the epithelial–mesenchymal transformation to leading to distant tissue colonization.^12^ Another study showed that miR-122-5p derived from exosomes of breast cancer cells can be taken up by stromal cells and down-regulate pyruvate kinase in cells, inhibiting glucose intake in the microenvironment and increase the available nutrients in the microenvironment of distant organs to promote distant metastasis of breast cancer cells.^13^ Therefore, we aimed to explore exosomal miRNAs that promote distant metastasis of breast cancer and the specific molecular mechanisms related to fibroblasts.

## 2. Results

### 2.1. Exosomal miRNA-stimulated fibroblasts promote migration of breast cancer cells

**Figure 1.**
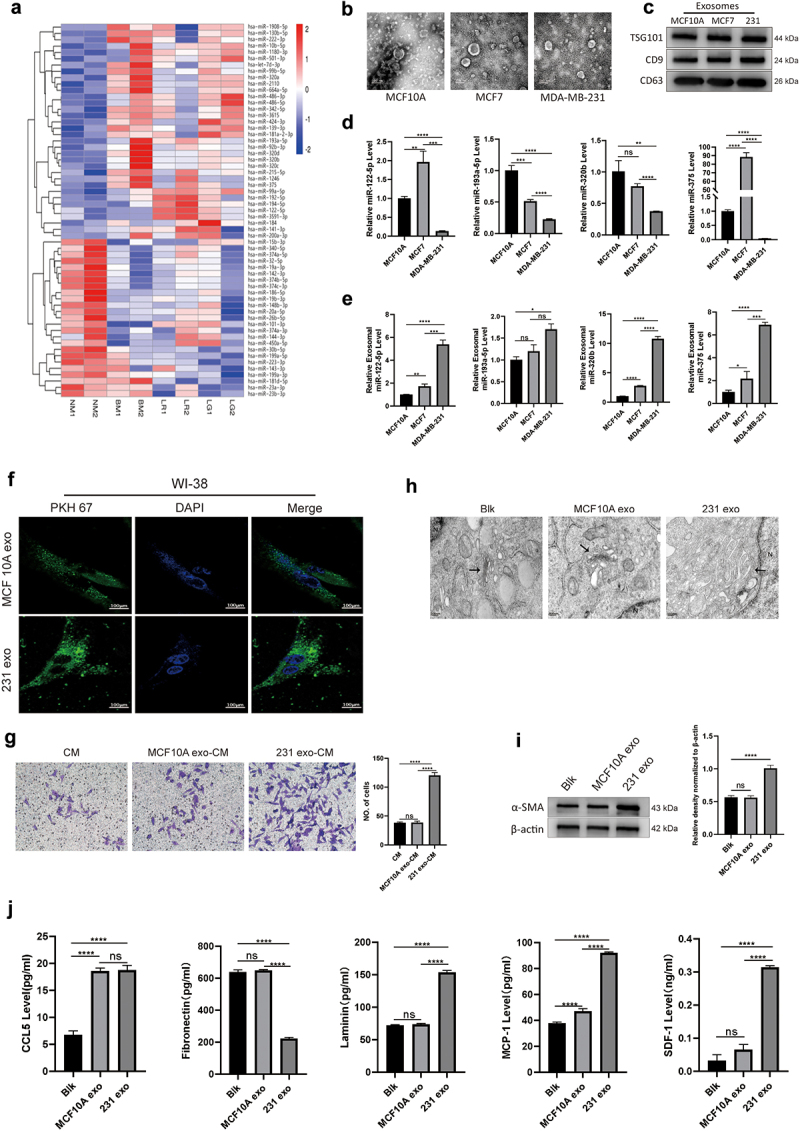
Exosomes-stimulated fibroblasts promote migration of breast cancer cells.

We collected plasma samples of breast cancer patients with different metastatic sites (lung, liver or bone) and those without metastasis, denoted as lung metastasis (LGM), liver metastasis (LRM), bone metastasis (BM) and no metastasis (NM). Exosomes purified from the plasma samples of these patients were subjected to RNA isolation and miRNA sequencing. We compared the raw miRNA expression profiling data between groups (LGM vs. NM, LRM vs. NM and BM vs. NM). The miRNAs profiles revealed significant difference between serum exosomes from breast cancer patients with different metastatic sites (Figure 1a). The up-regulated and down-regulated miRNAs were selected based on the >2-fold change and adjusted significant level (q value) between groups (LGM vs. NM, LRM vs. NM and BM vs. NM). Compared with NM group, there were 19 up-regulated miRNAs and 15 down-regulated miRNAs in LGM group, 11 up-regulated miRNAs and 3 down-regulated miRNAs in LRM group, 18 up-regulated miRNAs and 18 down-regulated miRNAs in BM group (Supplementary Table S3–8). Among these miRNAs, we selected several miRNAs (miR-122-5p, miR-193a-5p, miR-320b, and miR-375) related to cancer progress based on our study and previous reports.^14–17^ We isolated and purified exosomes from conditioned medium (CM) of the MCF10A, MCF7 and MDA-MB-231 cells. As shown in Figure 1b, exosomes appeared as discoid-like vesicles with a diameter of 30–150 nm by TEM. Exosomes specific proteins CD9, CD63 and TSG101 further verified that the isolated particles were exosomes (Figure 1c). The expression levels of miR-122-5p, miR-193a-5p, and miR-320b in MDA-MB-231 cells were significantly lower than in those in MCF10A cells (Figure 1d). However, the expression levels of miR-122-5p, miR-193a-5p, miR-320b, and miR-375 in exosomes derived from MDA-MB-231 cells were higher than those from MCF10A or MCF7 cells (Figure 1e). Next, WI-38 cells were co-cultured with MCF10A exosomes or MDA-MB-231 exosomes labeled with PKH67, and the results showed that a large number of exosomes were co-localized with WI-38 cells after PBS rinsing, indicating that WI-38 cells could internalize PKH67-labeled exosomes (Figure 1f). The CM of WI-38 cells treated with MDA-MB-231 exosomes could remarkably promote migration of MDA-MB-231 cells compared to MCF10A exosome treatment (Figure 1g). Electron microscopic images showed the hypertrophic Golgi pool in WI-38 cells after co-cultured with MDA-MB-231 exosomes (Figure 1h). Western blot analysis indicated that MDA-MB-231 exosomes increased α-SMA in WI-38 cells, suggesting the activation of WI-38 cells (Figure 1i). The supernatant of WI-38 cells incubated with exosomes from breast cancer cells could promote the migration of MDA-MB-231 cells (Figure 1g). Compared to MCF10A exosomes, MDA-MB-231 exosomes increased MCP-1, SDF-1, and laminin, but decreased fibronectin in CM of WI-38 cells (Figure 1j).

(a) This heat map showed 59 differentially expressed serum exosomal miRNAs in breast cancer patients with no metastases (NM, *n* = 2), lung metastases (LGM, *n* = 2), liver metastases (LRM, *n* = 2) and bone metastases (BM, *n* = 2). Intensity plots represented relative up-regulation (red) and down-regulation (blue) (*p* < .05). (b) Electron microscopic images of MDA-MB-231, MCF7, and MCF10A exosomes. Scale bar = 100 nm. (c) Western blot analysis of exosomes marker proteins of CD9, CD63, and TSG101. (d, e) The expression of miR-122-5p, miR-193a-5p, miR-320b, and miR-375 in cells (D) and exosomes (E) of MCF10A, MCF7, and MDA-MB-231 were detected by RT-qPCR. U6 served as internal references of miRNAs. (f) Representative images of PHK67-labeled MCF10A exosomes or MDA-MB-231 exosomes and WI-38 cells following co-culture (PKH67 presents green stains and DAPI presents blue stains). Scale bar = 200 μm. (g) Transwell migration analysis of MDA-MB-231 cells cultured with CM of WI-38 cells treated with MCF10A or MDA-MB-231 exosomes for 24 h. CM: conditional medium without treatment; MCF10A exo-CM: conditional medium of WI-38 cells treated with MCF10A exosomes; 231 exo-CM: conditional medium of WI-38 cells treated with MDA-MB-231 exosomes. (h) Electron microscopic images of WI-38 cells co-cultured with PBS (Blk group), MCF10A exosomes (10A exo group), or MDA-MB-231 exosomes (231 exo group). Arrows represented the Golgi apparatus and representative images were presented. (i) Western blot analysis of α-SMA in WI-38 cells treated with PBS (Blk group), MCF10A exosomes (10A exo group), or MDA-MB-231 exosomes (231 exo group), followed by the quantitative and statistical analysis results of proteins. (j) CCL5, MCP-1, SDF-1, laminin, and fibronectin expression in CM of WI-38 cells treated with PBS (Blk group), MCF10A exosomes (10A exo group) or MDA-MB-231 exosomes (231 exo group), 10A exo: MCF10A exosomes; 231 exo: MDA-MB-231 exosomes. **** *p* < .0001. Data (means ± standard deviations) between two groups were analyzed using unpaired t test. The experiment was repeated 3 times independently.

### 2.2. Exosomal miR-122-5p regulates secretory proteins expression in fibroblasts

**Figure 2.**
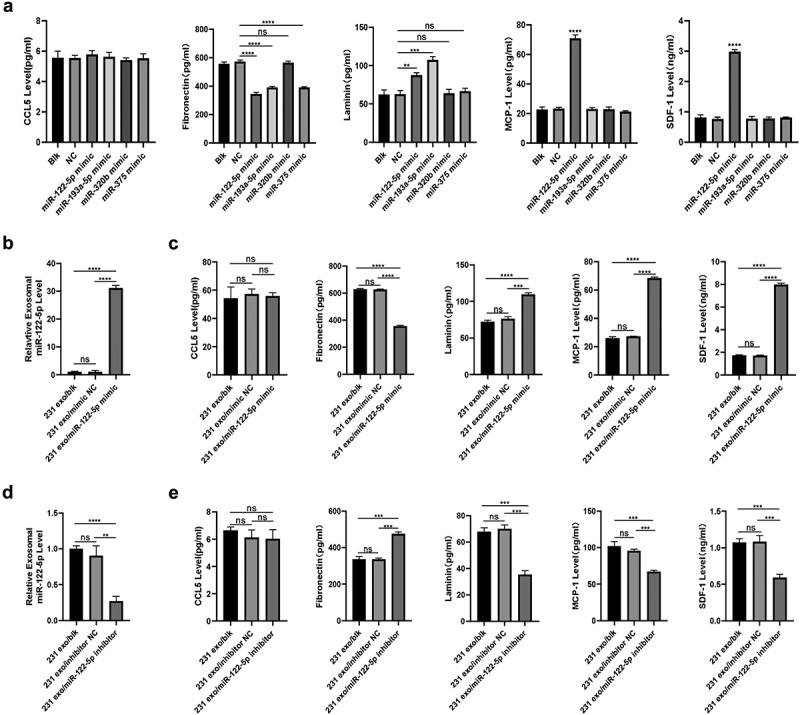
Exosomal miR-122-5p regulated the expression of secretory proteins from fibroblasts.

After overexpressing miR-122-5p, miR-193a-5p, miR-320b, or miR-375 with miRNA mimics, We found that miR-122-5p significantly increased MCP-1, SDF-1, and laminin, but decreased fibronectin in CM of WI-38 cells (Figure 2a). We overexpressed or knocked down miR-122-5p in MDA-MB-231 cells by transfecting miR-122-5p mimic or inhibitor, respectively, and then extracted exosomes from these cells (Figure 2b, d). MiR-122-5p overexpressed exosomes had no significant effect on the secretion of CCL5, but up-regulated MCP-1, SDF-1, and laminin, down-regulated fibronectin from WI-38 cells (Figure 2c). In addition, miR-122-5p knockdown exosomes showed the opposite effects on protein secretion by WI-38 cells (Figure 2e).

(a) CCL5, MCP-1, SDF-1, laminin, and fibronectin expression in CM of WI-38 cells treated with PBS, negative control (NC), miR-122-5p, miR-193a-5p, and miR-320b, miR-375 mimics were detected by ELISA analysis. (b) The overexpression efficiency of MDA-MB-231 cell exosomes carrying miR-122-5p mimic. (c) Effects of miR-122-5p overexpression on the secretion of CCL5, fibronectin, laminin, MCP-1, and SDF-1 in WI-38 cells. (d) The knockdown efficiency of MDA-MB-231 cells exosomes carrying miR-122-5p inhibitor. (e) Effects of miR-122-5p knockdown on the secretion of CCL5, fibronectin, laminin, MCP-1, and SDF-1 in WI-38 cells. *p<0.05; **p<0.01;***p<0.001; ****p<0.0001.

### 2.3. Exosomal miR-122-5p promotes migration of breast cancer cells by directly targeting MKP-2

**Figure 3.**
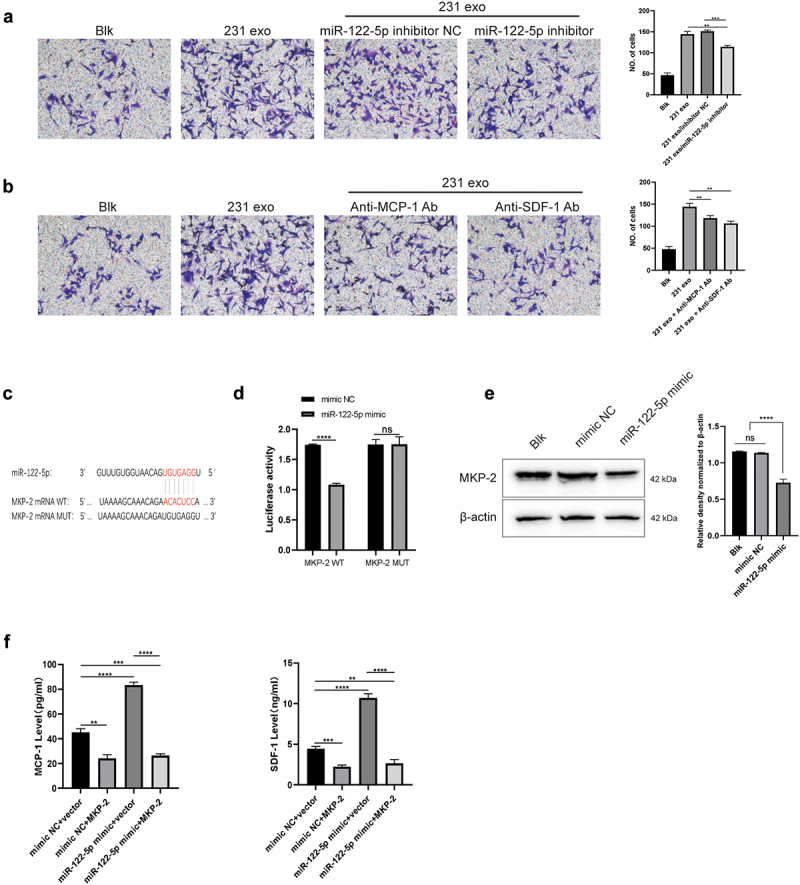
Exosomal miR-122-5p promotes migration of breast cancer cells by directly targeting MKP-2.

The transwell analysis showed that CM of WI-38 cells treated with MDA-MB-231 exosomes promoted the migration of MDA-MB-231 cells, but this effect was significantly down-regulated when miR-122-5p was knocked down in the exosomes (Figure 3a). Moreover, MCP-1 and SDF-1 neutralizing antibodies reduced the effect of exosomes on promoting MDA-MB-231 migration (Figure 3b). We compared the gene sequences of miR-122-5p and MKP-2 and found the binding sites of the two, and designed wild-type and mutant plasmids (Figure 3c). The luciferase activity was restrained in WI-38 cells co-transfected with miR-122-5p mimic and MKP-2-wild-type (WT), while it showed no significant changes in cells co-transfected with miR-122-5p mimic and MKP-2 mutant (MUT) (Figure 3d). To further verify this finding, miR-122-5p mimic was used to transfect WI-38 cells. MiR-122-5p overexpression led to reduced MKP-2 compared to NC (Figure 3e). To clarify the role of MKP-2 in secretion of MCP-1 and SDF-1, WI-38 cells were co-transfected with miR-122-5p mimic and MKP-2 plasmid. As shown in Figure 3f, compared with miR-122-5p mimic NC and MKP-2 plasmid, miR-122-5p mimic and MKP-2 plasmid-transfected WI-38 cells secreted less MCP-1 and SDF-1, suggesting miR-122-5p could up-regulated MCP-1 and SDF-1 by directly targeting MKP-2 in WI-38 cells.

(a) Transwell migration analysis of MDA-MB-231 cells cultured with CM of WI-38 cells treated with MDA-MB-231 exosomes or exosomes separated from MDA-MB-231 cells transfected with miR-122-5p inhibitor or negative control (NC) for 24 h. CM: conditional medium without treatment; 231 exo-CM: conditional medium of WI-38 cells treated with MDA-MB-231 exosomes; 231 exo/inhibitor NC-CM: conditional medium of WI-38 cells treated with exosomes derived from MDA-MB-231 cells transfected with miR-122-5p inhibitor NC; 231 exo/inhibitor-CM: conditional medium of WI-38 cells treated with exosomes derived from MDA-MB-231 cells transfected with miR-122-5p inhibitor. (b) Transwell migration analysis of MDA-MB-231 cells cultured with CM of WI-38 cells treated with MDA-MB-231 exosomes or MDA-MB-231 exosomes plus anti-MCP-1 neutralizing antibody or anti-SDF-1 neutralizing antibody. 231 exo-CM: conditional medium of WI-38 cells treated with MDA-MB-231 exosomes. (c) The binding sites between MKP-2 and miR-122-5p. (d) The binding of miR-122-5p to MKP-2 in WI-38 cells confirmed by the dual-luciferase reporter gene assay. MKP-2-WT: MKP-2 wild type; MKP-2-MUT: MKP-2 mutant. (e) Western blot analysis of MKP-2 in WI-38 cells transfected with miR-122-5p mimic and NC and the relative density normalized to β-actin. (f) MCP-1 and SDF-1 in CM of WI-38 cells co-transfected with miR-122-5p mimic or NC and MKP-2 or vector were detected by ELISA analysis. **p < 0.01; ***p < 0.001; ****p < 0.0001. The experiment was repeated 3 times independently.

### 2.4. Exosomal miR-122-5p promotes the establishment of PMN and breast cancer metastasis in vivo

**Figure 4.**
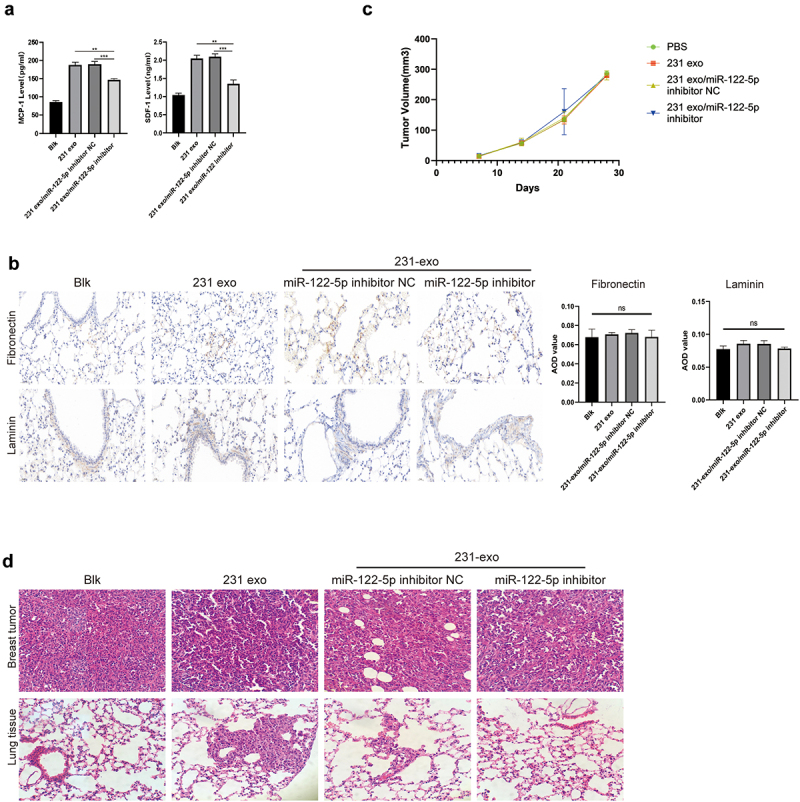
Exosomal miR-122-5p promotes the establishment of PMN and breast cancer metastasis in vivo.

We periodically injected exosomes into mice through tail vein and found that exosomes containing miR-122-5p could increase the expression levels of MCP-1 and SDF-1 in serum (Figure 4a). Immunohistochemistry showed that the expression of fibronectin and laminin in mouse lung tissue did not change after injection of exosomal miR-122-5p (Figure 4b). After 3 weeks of intravenous injection of exosomes, MDA-MB-231 cells were inoculated into mouse mammary gland tissue, and there was no significant difference of primary tumor growth among groups (Figure 4c). HE staining of primary tumor and lung tissue showed that the primary breast tumor cells displayed obvious nuclear heteromorphism and invasive growth, and tumor metastasis could be detected in lung tissue of mice injected with exosomal miR-122-5p or NC, but no metastasis was found in PBS group or exosomal miR-122-5p inhibitor group (Figure 4d).

(a) MCP-1 and SDF-1 levels in the serum of mice treated with exosomes derived from MDA-MB-231 cells for 3 weeks. (b) IHC staining of fibronectin and laminin in mouse lung tissues. Followed by the fibronectin and laminin AOD value of in lung tissues in four groups. ns, no significance. (c) The growth curves of primary xenograft tumor of mice. (d) HE staining of primary breast tumors and lung tissues after the establishment of orthotopic tumor model for 1 month. The magnification of pathological sections was 400 times. **p < 0.01; ***p<0.001.

## 3. Discussion

Breast cancer has become one of the most common female malignant tumors in the world. Invasiveness and metastasis are important features of malignant breast cancer. But there are still many uncertainties about how breast cancer cells metastasize to distant organs.

The production process of exosomes is related to the formation of multivesicular bodies (MVBs) in cells. MVBs are then fused to the plasma membrane and exocytosis, and exosomes are eventually secreted to the outside of the cells. Depending on the source cell type, environmental conditions and other factors, exosomes contain cellular components including DNA, RNA, lipids, metabolites, and cell surface proteins. These characteristics give the exosome composition heterogeneity and functional complexity.^18^ We compared the levels of serum exosomal miRNAs from breast cancer patients with different metastatic status and those from different breast cancer cells, and found that differential exosomal miRNAs screened from patients with metastasis have higher expression levels in exosomes secreted by MDA-MB-231 cells. The aggregation of exosome contents and the budding of exosome vesicles can occur through endosome sorting complex dependent and independent mechanisms.^19^

The selective loading mechanism endows exosomes with different characteristics, such as the propensity to transfer to certain organs and uptake by specific cell types. PKH67 is a lipophilic dye, which can bind to exosome membrane and emit fluorescence. In our experiment, WI-38 cells were incubated with PKH67 labeled exosomes from MCF10A and MDA-MB-231 cells. It was observed that WI-38 cells could internalize a large number of exosomes from MCF10A and MDA-MB-231 cells at low power under the fluorescence microscope, which confirmed the uptake of exosomes by WI-38 cells in vitro. The mode of exosome interaction with the cell surface of fibroblast and the mechanisms that mediate the transfer of exosome cargoes have not been fully elucidated. Exosomes interact with receptor cells mainly through the following ways: 1) Directly bind to cell receptors such as integrins, activation of recipient cell signaling pathways; 2) Fuse with the plasma membrane of the recipient cell, and the vesicle contents were transported to the cell to play biological functions; 3) Entry into the recipient cells by endocytosis, but the possibility of vesicle recycling and excretion of the recipient cells cannot be ruled out. Despite different contents and sizes, the principles of uptake and general intercellular trafficking of exosome are likely to be shared.^20–22^

Many studies have shown that exosomes play an important role in the development and metastasis of various types of tumors.^5,23,24^ One study showed that exosomal miR-500a-5p derived from cancer-associated fibroblasts promotes breast cancer cell proliferation and metastasis through targeting USP28.^25^ Another study found that MD231 breast cancer cells with highly metastatic ability secreted exosomes which promoted osteoclast differentiation and activation, which consequently accelerates reconstruct microenvironment for bone metastasis.^26^ In our study, the supernatant of WI-38 treated with exosomes from MDA-MB-231 cells significantly promoted the migration of breast cancer cells compared with that treated with PBS or MCF10A exosomes. These results demonstrated that MDA-MB-231 exosomes can activate normal fibroblasts, possibly altering their phenotype and function.

Activated fibroblasts can express some marker proteins, such as alpha-smooth muscle actin (α-SMA), fibroblast activation protein alpha (FAP), and fibroblast-specific protein 1 (FSP1). In our experiment, α-SMA expression in WI-38 cells was detected, and the activation effect of breast cancer exosomes on WI-38 cells was verified. It showed that MDA-MB-231 exosomes can increase laminin expression levels in WI-38, but decrease fibronectin, suggesting that these proteins may be involved in extracellular matrix remodeling.

Most secretory proteins are synthesized from eukaryotic endoplasmic reticulum-associated ribosomes and contain an amino-terminal signal peptide, called the lead sequence. The pilot sequence mediates the recognition of polypeptides by signal recognition particles (SRP) and their subsequent sorting into the endoplasmic reticulum (ER). The ER-Golgi Intermediate compartment is transmitted to the Golgi apparatus. In our study, we observed that MDA-MB-231 exosomes could lengthen the Golgi apparatus pool of WI-38 cells under transmission electron microscope after incubation with breast cancer exosomes. The ELISA assay results showed that the supernatant of WI-38 treated with MDA-MB-231 exosomes contained more chemokines MCP-1 and SDF-1 than that treated with PBS or MCF10A exosomes. Although the expression level of CCL5 was also significantly increased, MCF10A could also up-regulate the expression of CCL5, suggesting that CCL5 may not be the main specific molecule in the invasion process.

MiRNAs can be transported by vesicle or protein carrier by various cells output and input, when they are transported to the receptor cells, they can still maintain physiological functions and thus change the behavior of the cells, becoming the medium of intercellular information transmission. Some studies have shown that miRNAs can affect the metastasis of cervical squamous cell carcinoma^27^ and colorectal cancer.^28^ MiR-122-5p mimics enhanced TNBC cell viability, proliferation, and invasion, and they promoted epithelial–mesenchymal transition by suppressing charged multivesicular body protein 3 through MAPK signaling.^29^ Knockdown of CDKN2B-AS1 affected miR-122-5p/STK39 axis and suppressed the progression of breast cancer.^30^ Crocin could inhibit proliferation of breast cancer cells through decreasing miR-122-5p expression and the subsequent increase of SPRY2 and FOXP2 expression.^31^ MiR-122-5p was shown to affect the expression of secretory proteins in WI-38 cells in our study. Therefore, we hypothesized that the exosomal miR-122-5p secreted by MDA-MB-231 could promote the secretion of chemokines MCP-1 and SDF-1 by fibroblasts in the PMN of distant organs. These chemokines reached the primary tumor site through blood circulation and bound to specific chemokine receptors on the surface of breast cancer cells, promoting the movement of cells to distant organs and tissues suitable for growth, leading to the spread and distant colonization of tumors. We then confirmed this hypothesis using neutralizing antibodies to MCP-1 and SDF-1, and found that MCP-1 and SDF-1 neutralizing antibodies significantly down-regulated the migration ability of MDA-MB-231 cells. So far, we demonstrated in vitro that MDA-MB-231 cell-derived exosomes carry high levels of miR-122-5p, which are taken up by CAFs, and the latter secrete more chemokines MCP-1 and SDF-1. These chemokines increased the migration ability of MDA-MB-231 cells. Exosomal miR-122-5p up-regulated MCP-1 and SDF-1 in mice serum and promote lung metastasis of breast cancer in vivo. Teng F et al. reported that CAFs derived from endometrial cancer tissues promoted endometrial cancer progression via SDF-1α/CXCR4 axis to activate PI3K/Akt and MAPK/Erk signalings in a paracrine-dependent manner.^32^

In the innate immune response, MKPs can regulate the vitality of innate immune cells and the function of antigen-presenting cells, and play an important role in regulating inflammation and the production of anti-inflammatory cytokines. In the adaptive immune response, MKPs can regulate cytokine biosynthesis and cell proliferation.^33^ As an important cytokine in the immune response, chemokines are likely to be regulated by MKPs. Study from Kim HS et al. identified that the loss of MKP-1 activity is a critical step in monocyte priming and the metabolic stress-induced conversion of blood monocytes into a proatherogenic phenotype.^34^ Our study confirmed that exosomal miR-122-5p could regulate MCP-1 and SDF-1 secretion by targeting MKP-2 in CAFs. The mechanisms of signal transduction and secretory regulation still need further experimental exploration,

Combined with these results, exosomal miR-122-5p derived from breast cancer cells could transform a normal fibroblast into CAFs. In CAFs, miR-122-5p increased the secretion of chemokines MCP-1 and SDF-1 by directly targeting MKP-2. These MCP-1 and SDF-1 enhanced the migration of breast cancer cells by binding to specific receptors on the cell surface, thereby promoting the metastasis of breast cancer.

## 4. Materials and methods

### 4.1. Patients and plasma samples

Plasma samples were collected from female breast cancer patients with or without metastasis in Cancer Hospital of Harbin Medical University, Harbin, China. In the metastatic groups, newly diagnosed metastases were confirmed by biopsy or imaging, and these metastases were limited in a single organ (lung, liver, or bone). No signs of metastase were found in routine inspection after breast cancer surgery in non-metastatic group. Eight enrolled patients did not receive chemotherapy, radiotherapy, or endocrine therapy before sampling. The collected plasma samples were stored at −80°C until use. The study was approved by the Research and Ethical Committee of Cancer Hospital of Harbin Medical University, and obtained the informed consent of all patients.

### 4.2. Cell culture

Normal breast mammary epithelial cell line MCF-10A and breast cancer cell lines (MCF-7, and MDA-MB-231) were purchased from Cell Bank of Shanghai Institute of Cells, Chinese Academy of Science (Shanghai, China). MCF-10A cells were cultured in MEGM BulletKit (Lonza, Switzerland) with 10% fetal bovine serum (FBS, Thermo Fisher Scientific, Waltham, MA, USA). MCF-7 and MDA-MB-231 cells were cultured in DMEM (GIBCO, Grand Island, NY, USA) containing 10% FBS and 1% penicillin/streptomycin (GIBCO, Grand Island, NY, USA). WI-38 human lung fibroblast cells were cultured in MEM (GIBCO, Grand Island, NY, USA) containing 10% FBS and 1% penicillin/streptomycin. All cells were maintained in a humidified incubator with 5% CO2 at 37°.

### 4.3. Exosome isolation

When the cell density reached 80%−90%, the medium was replaced with serum-free medium and continued to culture for 48 h. The cell supernatants were collected and centrifuged at 500 g for 15 min 12,500 g for 20 min at 4°C to remove cell debris. Next, to collect the exosome pellets, the supernatants were centrifuged at 110,000 g at 4°C for 2 h. Subsequently, the pellets were washed with PBS and subjected to a further round of ultracentrifugation at 110,000 g at 4ºC for 2 h. After removal of the supernatants, the exosome pellets were resuspended with 200 µL PBS and stored at − 80°C until further processing. In addition, the plasma exosomes were isolated using ExoQuick precipitation kit (SBI, USA) according to the manufacturer’s instructions.

### 4.4. Transmission electron microscopy (TEM)

For TEM analysis, exosomes were plated on formvar carbon-coated TEM grids for 5 min, and stained by 1% uranyl acetate for 10 min at room temperature 20–25°C. Then, the grids were washed three times with PBS and semi-dried with filter paper. WI-38 cells were washed with PBS and centrifuged at 500 g for 5 min to obtain a visible pellet. Cells were then fixed with a 1% glutaraldehyde and 0.2 M Hepes. The samples were further observed using a TEM (H7650, Hitachi, Tokyo, Japan).

### 4.5. Western blot

Cells or exosomes were fully lysed with RIPA (Lablead, Beijing, China), and the protein concentration was quantified using Bradford assay kit (Thermo Fisher Scientific, Waltham, MA, USA). Proteins were separated with sodium dodecyl sulfate-polyacrylamide gel electrophoresis (SDS-PAGE) and transferred onto PVDF membranes (Millipore, Billerica, MA, USA). The membranes were blocked with 5% skimmed milk for 1 h at room temperature 20–25°C and incubated with primary antibodies overnight at 4°C. Thereafter, the membranes were washed 3 times using TBST and incubated with HRP-conjugated secondary antibodies for 1 h at room temperature. We used the following primary antibodies: anti-CD9 (sc -13,118, Santa Cruz Biotechnology), anti-CD81 (sc -166,029, Santa Cruz Biotechnology), anti-TSG101 (sc-7964, Santa Cruz Biotechnology), α-SMA (ab5831, Abcam), MKP-2 (#5149, Cell Signaling Technology).

### 4.6. Fluorescent labeling exosomes

The PKH67 green fluorescence kit (Sigma-Aldrich) was used to label the purified exosomes. The exosomes were suspended in 1 mL Diluent C solution, and 4 μL PKH67 ethanol dye solution was added to 1 mL Diluent C to prepare dye solution. The above solutions were gently mixed and placed in the dark at room temperature for 5 min. The staining was terminated with 1% BSA for 1 min. The labeled exosomes were ultracentrifuged for 70 min at 100,000 × g, washed with PBS, ultracentrifuged again, and resuspended in 200 μL PBS. The PKH67-labeled exosomes were incubated with WI-38 cells at 37°C for 12 h. The cells were then fixed with 4% paraformaldehyde and the nucleus were stained with the DAPI. The uptake of exosomes by WI-38 cells was observed under microscope.

### 4.7. Transwell migration assay

Each upper chamber was seeded with 1 × 10^5^ MDA-MB-231 cells in 100 μL of serum-free medium, and incubated with WI-38 cell supernatants treated with MCF10A exosomes, MDA-MB-231 exosomes or exosomes collected from MDA-MB-231 cells transfected with miR-122-5p inhibitor or negative control (NC), and 500 μL medium containing 10% FBS was added into lower chamber. After 24 h, the chambers were fixed with 4% paraformaldehyde (PFA, Biosharp, Anhui, China) for 30 min, stained with 0.5% crystal violet (Solarbio, Beijing, China) solution for 10 min and washed three times with PBS. Cells in the upper chambers were removed using a cotton swab. Transmembrane cells were counted in three random fields.

### 4.8. ELISA assay

ELISA analysis was performed according to the reagent manufacturer’s instructions. Add 100 μL WI-38 cell supernatants or diluted mice serum and standard protein to the wells of 96-well plate and incubated at room temperature for 2 h. After washing off the remaining liquid, add diluted primary antibody and incubate at room temperature for 1 h, followed by adding diluted HRP-labeled secondary antibody and incubating at room temperature for 40 min. Next, TMB was added to each well and incubated for 20 min at 37°C in the dark. Then the stop solution was added to wells and the 96-well plate was immediately detected using the microplate reader at 450 nm. The antibody concentration in the cell supernatants was calculated according to the standard curve.

### 4.9. RNA extraction and quantitative real-time PCR (RT-qPCR)

The total RNA was extracted with a RNeasy Mini kit (QIAGEN, Valencia, CA, USA). Extracted RNA was then reverse transcribed into complementary DNA (cDNA) using the reverse transcription kit (Takara, Shiga, Japan). RT-qPCR was conducted on an ABI 7500 instrument (Applied Biosystems, Foster City, CA, USA). U6 served as internal references of miRNA. The fold changes were calculated using relative quantification (the 2−ΔΔCt method). The primer sequences used in our study were listed in Supplementary Table S1.

### 4.10. Cell transfection

According to the instructions of Lipofectamine 2000 transfection reagent, MDA-MB-231 cells at logarithmic phase were seeded in three 6-well plates (5 × 10^5^ cells per well). Then, 25 pmol miR-122-5p mimic/inhibitor and NC as well as 10 μL transfection reagent were added to each well (10 pmol/mL). To investigate the effect of MKP-2 on the function of WI-38 cells treated with exosomes or miR-122-5p mimic, MKP-2 plasmid and the vector were used to transfect WI-38 cells. The cells were cultured for 48 h continuously and used for further experiments. The sequences of miRNA mimic/inhibitor referred above were listed in Supplementary Table S2.

### 4.11. Dual-luciferase reporter gene assay

WI-38 cells were cultured in a 48-well plate for 24 h. The plasmids were used to construct the plasmids of MKP-2-WT-3′ untranslated region (3′ UTR) and MKP-2-MUT-3′ UTR. Next, the constructed plasmids were co-transfected with 50 nM miR-122-5p mimic or NC to WI-38 cells for 48 h. The Dual-Luciferase Reporter Assay System (Promega, Madison, WI, USA) was used to detect the ratio of renilla luciferase activity to firefly luciferase activity and to verify the direct target gene of miR-122-5p.

### 4.12. Orthotopic xenograft models and histological analysis

All animal studies were undertaken in accordance with the NIH Guide for the Care and Use of Laboratory Animals, with the approval of the Laboratory Animal Management and Ethics Committee of Xiamen University. To evaluate the effect of MDA-MB-231 derived exosomal miR-122-5p on tumor metastasis, female Balb/c nude mice (aged 4–6 weeks) were randomly separated into four groups (*n* = 10). In the four groups, 30 µg MDA-MB-231 exosomes, MDA-MB-231 exosomes transfected with miR-122-5p inhibitor, MDA-MB-231 exosomes transfected with inhibitor negative control or PBS, were intravenously injected into nude mice through the tail vein every 3 days, respectively. After 3 weeks, half of these mice were sacrificed to collect serum for ELISA analysis and excise lung tissues for immunohistochemistry analysis. For the other half of the mice, 5 × 10^6^ MDA-MB-231cells were injected into the fourth pair of mammary glands on the right side. Tumor volume (length × width^2^  × 0.5) was recorded every 7 days for 4 weeks. After 4 weeks, the mice were sacrificed, breast tumors and lung tissues were incubated overnight in 4% paraformaldehyde for hematoxylin–eosin (HE) staining.

### 4.13. Immunohistochemical staining

Lung tissues harvested from mice were immersion-fixed in 4% paraformaldehyde for 24 h, embedded in paraffin and sectioned at 5 μm. Five sections in each group were dewaxed, hydrated, and incubated with anti-fibronectin (ab2413, Abcam) and anti-laminin (ab11575, Abcam) overnight at 4°C and then probed with horse radish peroxidase (HRP)-conjugated secondary antibody (ab205718, Abcam) at room temperature for 1 h. Five sections in each group were stained using diaminobenzidine (DAB), counterstained by hematoxylin for 1 min, observed and photographed under a microscope.

### 4.14. Statistical analysis

Continuous variables were described as mean ± standard deviation, and were compared using ANOVA for normally distributed variables, or the Kruskal – Wallis rank test for non-normally distributed variables, and the χ2 test for categorical variables. SPSS 25.0 and GraphPad Prism 8 software were used for statistical analysis and plotting.

## Supplementary Material

supplementary table.docx

## Data Availability

All data generated or analyzed during this study are included in this article and its supplementary file. Further enquiries can be directed to the corresponding author.
