# Supplementary material for: Exosomal miR-122-5p for regulation of secretory functions of fibroblasts and promotion of breast cancer metastasis by targeting MKP-2: an experimental study
Source: Cancer Biol Ther. 2025 May 4;26(1):2500104. doi: 10.1080/15384047.2025.2500104 (PMC12051585; doi:10.1080/15384047.2025.2500104)
Supplement: supplementary table.docx [file KCBT_A_2500104_SM9615.docx]

Supplementary Information

Supplementary Table S1. Primer sequences used in this study

|  | Sense primer (5’-3’) | Antisense primer(5’-3’) |
| --- | --- | --- |
| miR-122-5p | AACGCCATTATCACACTAAATA | AGTGCAGGGTCCGAGGTATT |
| miR-193a-5p | TATATGGGTCTTTGCGGGCG | GTGCAGGGTCCGAGGT |
| miR-320b | TCCGAAACGGGAGAGTTGG | GTGCAGGGTCCGAGGT |
| miR-375 | CGCGCTTTGTTCGTTCGGCTC | ATCCAGTGCAGGGTCCGAGG |
| U6 | CTCGCTTCGGCAGCACA | AACGCTTCACGAATTTGCGT |

Supplementary Table S2. Sequences of miRNAs mimics and inhibitors.

|  | Sense primer (5’-3’) | | Antisense primer(5’-3’) |
| --- | --- | --- | --- |
| miR-122-5p mimic | CAAACACCAUUGUCACACUCCA | CACAAAUUCGGUUCUACAGGGUA | |
| mimics NC | UUUGUACUACACAAAAGUACUG | CAGUACUUUUGUGUAGUACAAA | |
| miR-122-5p inhibitor | UGGAGUGUGACAAUGGUGUUUG |  | |
| inhibitor NC | CAGUACUUUUGUGUAGUACAAA |  | |

Supplementary Table S3. Up-regulated miRNA of breast cancer patients with lung metastases.

| miRNA | Fold change | Adjusted P-value |
| --- | --- | --- |
| hsa-miR-320b | 2.0179 | 0.000000034598 |
| hsa-miR-320c | 1.9243 | 0.0013334 |
| hsa-miR-10b-5p | 1.4864 | 0.0020238 |
| hsa-miR-1180-3p | 1.9848 | 0.0022621 |
| hsa-miR-486-3p | 1.8535 | 0.0029593 |
| hsa-miR-486-5p | 1.8259 | 0.0029862 |
| hsa-miR-193a-5p | 1.7427 | 0.0065798 |
| hsa-miR-320a | 1.2436 | 0.0097868 |
| hsa-miR-3615 | 1.6332 | 0.012633 |

| hsa-miR-184 | 2.0643 | 0.015311 |
| --- | --- | --- |
| hsa-miR-2110 | 1.3973 | 0.015735 |
| hsa-miR-375 | 1.9793 | 0.015735 |
| hsa-miR-501-3p | 1.6559 | 0.016952 |
| hsa-miR-130b-5p | 1.3409 | 0.021903 |
| hsa-miR-424-3p | 1.3268 | 0.021903 |
| hsa-miR-99a-5p | 1.3044 | 0.021903 |
| hsa-miR-181a-2-3p | 1.2366 | 0.025384 |
| hsa-miR-139-3p | 1.3309 | 0.026554 |
| hsa-miR-1908-5p | 1.1603 | 0.032627 |

Supplementary Table S4. Down-regulated miRNA of breast cancer patients with lung metastases.

| miRNA | Fold change | Adjusted P-value |
| --- | --- | --- |

| hsa-miR-223-3p | -2.2816 | 0.0000066872 |
| --- | --- | --- |
| hsa-miR-199a-5p | -1.436 | 0.000131 |
| hsa-miR-23a-3p | -1.8936 | 0.000797 |
| hsa-miR-374b-5p | -1.6984 | 0.001333 |
| hsa-miR-374c-3p | -1.6984 | 0.001333 |
| hsa-miR-30b-5p | -1.6643 | 0.002024 |
| hsa-miR-142-3p | -1.4622 | 0.010239 |
| hsa-miR-199a-3p | -1.2417 | 0.025384 |
| hsa-miR-19a-3p | -1.6797 | 0.025946 |
| hsa-miR-23b-3p | -1.2928 | 0.026554 |
| hsa-miR-143-3p | -0.91692 | 0.030283 |
| hsa-miR-19b-3p | -1.6688 | 0.030602 |
| hsa-miR-374a-5p | -1.4241 | 0.032627 |
| hsa-miR-181d-5p | -1.0744 | 0.040513 |

Supplementary Table S5. Up-regulated miRNA of breast cancer patients with liver metastases.

| miRNA | Fold change | Adjusted P-value |
| --- | --- | --- |
| hsa-miR-122-5p | 3.016 | 0.0000044384 |
| hsa-miR-3591-3p | 3.016 | 0.0000044384 |
| hsa-miR-193a-5p | 2.5401 | 0.000082199 |
| hsa-miR-375 | 2.5112 | 0.004477 |
| hsa-miR-192-5p | 1.484 | 0.020706 |
| hsa-miR-1246 | 2.1956 | 0.020907 |
| hsa-miR-200a-3p | 2.077 | 0.020907 |
| hsa-miR-99a-5p | 1.471 | 0.020907 |
| hsa-miR-194-5p | 1.4433 | 0.032879 |
| hsa-miR-320b | 1.366 | 0.032879 |
| hsa-miR-141-3p | 1.9399 | 0.046714 |

Supplementary Table S6. Down -regulated miRNA of breast cancer patients

with liver metastases.

| miRNA | Fold change | Adjusted P-value |
| --- | --- | --- |
| hsa-miR-223-3p | -1.4797 | 0.020907 |
| hsa-miR-374b-5p | -1.407 | 0.044935 |
| hsa-miR-374c-3p | -1.407 | 0.044935 |

Supplementary Table S7. Up-regulated miRNA of breast cancer patients with

bone metastases.

| miRNA | Fold change | Adjusted P-value |
| --- | --- | --- |
| hsa-miR-375 | 3.8133 | 0.0000093857 |
| hsa-miR-1246 | 3.4059 | 0.000227 |
| hsa-miR-130b-5p | 1.7914 | 0.000527 |
| hsa-miR-3615 | 1.7045 | 0.000527 |
| hsa-miR-320d | 3.1446 | 0.000535 |
| hsa-miR-320c | 2.5891 | 0.007667 |
| hsa-miR-99b-5p | 1.3931 | 0.007667 |
| hsa-miR-320b | 2.4871 | 0.007827 |
| hsa-miR-193a-5p | 2.3865 | 0.01414 |
| hsa-miR-215-5p | 1.8889 | 0.020496 |
| hsa-miR-200a-3p | 2.0224 | 0.030969 |
| hsa-miR-222-3p | 1.1914 | 0.030969 |
| hsa-miR-320a | 1.4224 | 0.030969 |
| hsa-miR-342-5p | 1.2891 | 0.030969 |
| hsa-let-7d-3p | 1.0869 | 0.031925 |
| hsa-miR-2110 | 1.4388 | 0.041622 |
| hsa-miR-664a-5p | 1.3121 | 0.041622 |
| hsa-miR-92b-3p | 1.8532 | 0.041622 |

Supplementary Table S8. Down-regulated miRNA of breast cancer patients

with bone metastases.

| miRNA | Fold change | Adjusted P-value |
| --- | --- | --- |
| hsa-miR-101-3p | -1.6506 | 0.000227 |
| hsa-miR-374b-5p | -2.0297 | 0.002271 |
| hsa-miR-374c-3p | -2.0297 | 0.002271 |
| hsa-miR-20a-5p | -1.2261 | 0.01184 |
| hsa-miR-148b-3p | -1.1099 | 0.013877 |
| hsa-miR-30b-5p | -1.4828 | 0.014042 |
| hsa-miR-19b-3p | -1.9504 | 0.016299 |
| hsa-miR-142-3p | -1.7618 | 0.018944 |
| hsa-miR-340-5p | -1.773 | 0.020496 |
| hsa-miR-450a-5p | -1.668 | 0.020496 |
| hsa-miR-374a-5p | -1.7646 | 0.022523 |
| hsa-miR-144-3p | -1.9729 | 0.030969 |
| hsa-miR-26b-5p | -1.2689 | 0.030969 |
| hsa-miR-32-5p | -1.355 | 0.030969 |
| hsa-miR-186-5p | -1.2633 | 0.031925 |
| hsa-miR-19a-3p | -1.8099 | 0.038015 |
| hsa-miR-15b-3p | -1.3673 | 0.041622 |
| hsa-miR-374a-3p | -1.5168 | 0.041622 |
